# Exploring Mental Health Presentations in Remote Aboriginal Community Controlled Health Services in the Kimberley Region of Western Australia Using an Audit and File Reviews

**DOI:** 10.3390/ijerph19031743

**Published:** 2022-02-03

**Authors:** Emma Carlin, Zaccariah Cox, Kristen Orazi, Kate L. Derry, Pat Dudgeon

**Affiliations:** 1 Kimberley Aboriginal Medical Services, Broome, WA 6725, Australia; sewbresearchofficer@kamsc.org.au; 2 The Rural Clinical School of Western Australia, The University of Western Australia, Broome, WA 6725, Australia; execmentalhealth@kamsc.org.au; 3 School of Indigenous Studies, University of Western Australia, Perth, WA 6009, Australia; kate.derry@uwa.edu.au (K.L.D.); pat.dudgeon@uwa.edu.au (P.D.)

**Keywords:** Aboriginal, Indigenous, mental health, Aboriginal community controlled health services

## Abstract

The study aims to explore the role of mental health care in remote Aboriginal health services in the Kimberley region of Western Australia and provide a more nuanced understanding of the patients presenting for care, their needs, and the clinical response. Little is currently known about primary health care presentations for mental health, suicide, and self-harm for remote dwelling Aboriginal residents of the Kimberley region, despite high rates of psychological distress, self-harm, and suicide across the area. This study was progressed through a retrospective, cross-sectional audit of the electronic medical records system used by three remote clinics to explore the interactions recorded by the clinics about a patient’s mental health. In addition, an in-depth file review was conducted on a stratified purposive sample of 30 patients identified through the audit. Mental ill-health and psychological distress were found to be prominent within clinical presentations. Psychosocial factors were frequently identified in relation to a patient’s mental health presentation. Optimizing patients’ recovery and wellness through service improvements, including an enhanced mental health model of care, is an important next step.

## 1. Introduction

The Aboriginal and Torres Strait Islander people (First Nations people) of Australia have survived colonisation and continue to survive its sequelae of marginalisation, dispossession, and racism. First Nations people across Australia are resilient and, in many cases, flourishing; however, at a population level, there is psychological distress [1]. Self-reported surveys [2], hospitalizations with mental health as the primary reason for admission [3], suicide rates [4], and self-harm data [5] all demonstrate the high rates of mental ill-health amongst the First Nations population. Reducing rates of mental ill-health, including suicide, amongst First Nations people is recognized by the Australian Government as a matter of national priority [6]. Ensuring responses are governed by First Nations people, aligned to a First Nation framework of health [7], and responsive to the context of trauma and social disadvantage is an ongoing process of advocacy and change [8].

First Nation Australians have experienced a range of barriers that have inhibited engagement with mental health support and services. These include limited knowledge of the mainstream mental health paradigm, parallel cultural beliefs, shame, fear of the consequences of a diagnosis (e.g., removal of children), and inequitable access to culturally secure care [9,10,11,12,13]. Primary health care services, including Aboriginal Community Controlled Health Services (ACCHS) [14], are increasingly engaging and responding to the mental ill-health of First Nations people [15].

There is little scholarly investigation into the interaction between primary health care and mental health care for First Nation Australians in remote Australia. This is despite the rates of mental ill-health being much higher in geographically remote communities [16]. Remote Australia has a paucity of specialist mental health and therapeutic services [15,17] which further emphasizes the centrality of primary health in providing mental health care. This study examined data from the electronic medical records of three remote primary health care clinic sites, all ACCHS, in the Kimberley region of Western Australia. The aim of the study is to explore the role of mental health care in remote ACCHS and provide a more nuanced understanding of the patients presenting for care, their needs, and the clinical response.

## 2. Materials and Methods

### 2.1. Setting

The Kimberley region is a remote and expansive region of Western Australia, some 2000 km north of Perth, the state capital. The Kimberley has a population of about 34,000 people, with approximately 42% of all residents identifying as Aboriginal [18]. At a population level, Aboriginal residents of the Kimberley experience poorer health outcomes than non-Aboriginal residents [19].

The remote communities that are reported on in this study have their primary health care provided by an ACCHS. These ACCHSs all have emergency care capacity with the nearest tertiary health service between 170 and 300 km away. The Kimberley Mental Health and Drug Service (KMHDS), a Western Australian government service, provides a regular visiting mental health specialist schedule to each of the communities included in this study. Eligible patients have care shared between their ACCHS and KMHDS, allowing KMHDS to enter progress notes in the patients ACCHS electronic medical record.

The communities in this study have been de-identified and are referred to as Clinic 1, 2, and 3. The adult population of the communities included in this study ranges from approximately 170 to 350 people [20].

### 2.2. Study Design

This study was progressed through a retrospective, cross-sectional audit of the electronic medical records system (MMEx, ISA Health Care 2021) used by three remote Kimberley clinics to explore the interactions recorded by the clinics about a patient’s mental health. We conducted a wildcard search for mental*; self-harm*; suicid* for all patients aged 18 years who identified as Aboriginal and accessed the clinics at least once during the audit period. These search terms were informed by conversations with six local clinicians who believed that these terms were best placed to progress the aims of the audit. The audit covered twelve months between 1 January 2020 and 31 December 2020. Search terms applied to the following MMEx fields: “diagnosis”, “primary presenting complaint”, and “other presenting complaint”. Information input into these categories consists of free text and/or use of pre-existing clinical codes. Information was also collected from the inbuilt suicide and self-harm screening questions, consisting of six structured questions with predefined answers to assess a patient’s suicide ideation, plan, intention, and self-harm status.

The term interaction refers to any entry in the patient’s electronic medical record that includes the above search terms. This includes all progress notes recorded on the patient’s MMEx record by ACCHS and KMHDS staff. Data were collected in Excel spreadsheets, cleaned, and descriptively analyzed using Stata statistical software version 17 (StataCorp, 2021, College Station, TX, USA). Data analysis explored the number of patients, their age, sex, clinic site, type of interaction, and number of interactions per patient over the audit period.

In addition to the audit, an in-depth file review was conducted on a stratified purposive sample of 30 patients identified through the audit (10 with 1 coded interaction, 10 with 2–9 interactions, and 10 with >=10 interactions). Each sample of 10 prioritized gender parity, varied age, and a spread of clinic locations. The file review explored the type of mental health presentation, record of clinical care, record of diagnosis by a general practitioner or psychiatrist (if any), known alcohol or other drug status, presence of other psychosocial risk factors, and codes used to record a mental health interaction. Illustrative quotes selected by authors EC and ZC are used to provide a qualitative descriptive [21] exploration of the patient experiences as reflected through their MMEx file.

This study was supported by the Kimberley Aboriginal Health Planning Forum Research Subcommittee and the Western Australian Aboriginal Health Ethics Committee (Project 2020:017).

## 3. Results

### 3.1. Audit

A total of 92 patients comprising 403 clinical interactions were identified using the audit terms (Table 1). Gender was evenly represented in the data, but younger people (aged 18–34) were more pronounced, representing nearly 62% of the total audit population. Nearly half (45%) of all patients represented in the audit had one recorded interaction relating to mental health. The most commonly coded terms were “mental health problem” and “mental health disorder”.

### 3.2. File Review

File review patients are reported on in relation to audit stratification. Category A refers to patients with 1 coded interaction as found through the audit; Category B refers to 2–9 interactions; Category C refers to 10 or more interactions. The audit data identified only two women and no patients from Clinic 2 within Category C. Our file review sampling reflects this (Table 2).

#### 3.2.1. Clinical Coding

The number of coded interactions found in the audit was substantially less than the relevant interactions found in the file review (Table 3). This finding was most pronounced for Category C (10 or more interactions), with the median number of interactions four times greater using the file review method. The discrepancy in numbers relates to the health professionals’ coding of mental health presentations. Common characteristics of mental health coding are shown in Table 4. The file review revealed a wider constellation of mental health coding terms, including “agitation”, “review”, “follow up”, “personal administration”, and “medication given”. In some instances, the name of the medication provided was used to code the event. Presentations in which mental health matters were discussed alongside other health matters were often coded as “multiple complaints” or the coding prioritized the physical health matters such as “wet cough”, “wound management”, or “diabetes”. This was particularly evident in the files of patients who had complex health profiles (e.g., patients with a record of a significant morbidity and/or multi-morbidities concurrent to presentations relating to, and/or diagnosis of, mental health).

#### 3.2.2. Complex Health Profile

Complex health profiles were recorded for 12 of the 30 reviewed patients (Table 5). Complex health patients were identified through the MMEx alerts, labels, and diagnosis ribbon. Coronary artery disease and diabetes were the most commonly recorded primary health conditions. Examples of complex health profiles include:

Male patient aged 45–54: coronary artery disease, diabetes, dyslipidaemia, gastroesophageal reflux, hypercholesterolemia, hypertension, latent syphilis, low iron.

Female patient aged 35–44: Cirrhosis of liver, diabetes, history of heart failure, cervical screen abnormality, prosthetic mitral valve and tricuspid valve, replacement of the aortic valve, rheumatic heart disease (Priority 1/Severe).

**Table 5:** Characteristics of patients and clinical response for file review patients during 2020.

Documented Characteristics for File Review Patients	Patient Category Based on Number of Coded Mental Health-Related Interactions ^a^			
	A (1 Coded) *n* = 10	B (2–9) *n* = 10	C (≥10) *n* = 10	Total *n* = 30
**Patient characteristics and external stakeholder involvement in 2020**				
Alcohol or other drug use documented				
No	3	4	2	9
Yes	7	6	8	21
Psychosocial factors ^b^				
Family violence ^c^	5	4	1	10
Other violence	1	1	0	2
Other family and/or partner conflict	1	5	3	9
Assault of family member	2	3	3	8
Imprisonment	0	1	1	2
Greif and loss	2	2	1	5
Food insecurity	2	0	0	2
Under guardianship order	0	0	4	4
Overcrowding/insufficient housing	2	2	5	9
Complex primary health	6	5	1	12
None	0	1	3	4
External stakeholders involved in relation to mental health care ^b^				
Tertiary health provider	3	6	2	11
Specialist mental health service	6	6	9	21
Allied health (counselling/psychological services)	3	1	0	4
Alcohol and Other Drug services	0	0	1	1
Police	4	1	0	5
Other	1	0	1	2
None	1	3	0	4
**Characteristics of clinical response in 2020**				
Documentation of mental health screening				
No	7	8	10	25
Yes	3	2	0	5
Documentation of transfer to hospital due to mental health status				
No	7	4	8	19
Yes ^d^	3	6	2	11
Documentation of medication related to mental health				
No	8	4	0	12
Yes	2	6	10	18
Documentation of diagnosis				
No	9	4	0	13
Yes	1	6	10	17
Schizophrenia ^e^	0	3	9	12
Schizophreniform disorder	0	1	0	1
Organic psychosis	0	0	1	1
Post-traumatic stress disorder	1	0	0	1
Depression and/or anxiety	0	2	0	2
Documentation of follow up clinic care relating to distress/mental health				
No	6	4	0	10
Yes	4	6	10	20

^a^ Interactions relating to mental health indicated by progress notes in 2020 that included the terms “mental *”, “self-harm”, or “suicid *”. ^b^ Some patients had multiple psychosocial factors and/or external stakeholders documented. ^c^ Family violence includes Intimate Partner Violence. ^d^ 3 patients were transported by clinic and 6 by Royal Flying Doctor Service. ^e^ 4 out of 9 patients with schizophrenia in the ≥10 group had a relevant comorbidity, including intellectual disability, fetal alcohol spectrum disorder, antisocial personality disorder, and bipolar disorder.

#### 3.2.3. The Psychosocial Context of Mental Health Presentations

Family violence, family conflict, drug and alcohol use, and insufficient housing were the most commonly noted psychosocial stressors documented for all reviewed patients (Table 5). Psychosocial stressors were found to be multiple and recurrent and were often associated with the coded interaction of mental ill-health, suicide, or self-harm:

‘*Intentional MVA* [motor vehicle accident], *pt* [patient] *stated he wanted to end his life after conflict and argument with family. Pt states he was in a wrong skin relationship and has been forced to end the relationship. Pt* [patient] *heavily intoxicated. Minor lacerations*’.

(After-hours presentation, entered by Remote Area Nurse (RAN). Male patient, aged 18-24)

‘*BIB* [brought in by] *police. Found by family with rope threatening suicide. Very upset by his actions, caused serious injury to adult son–son flown to Perth for treatment 1 day ago. Pt intoxicated. No money in the house, no food, has abdominal pain*’.

(After-hours presentation, entered by RAN. Male patient 45–54)

Psychosocial factors were also evident as antecedents to episodes of mental ill-health, suicide, or self-harm. Within Category A (one interaction), 5 of the 10 patients reviewed had a clinic presentation up to four weeks prior to the mental health coded event in which one or more stressors or risks were identifiable. These included assault by a family member (5/5), assault of a family member (2/5), and experience of grief and loss (2/5).

**File excerpt-female patient aged 35–44**

Interaction prior to coded interaction:

‘*Assault by brother. Noted pt* [patient] *called police. Pt stated they had been drinking*’.

(Clinic-hours presentation, entered by RAN)

Coded interaction, 24 h later:

‘*Pt phoned threatening suicide. AHW* [Aboriginal Health Worker] *attends house with me. Pt did not answer the door. Police called. Pt bought to the clinic. Stated she has no fridge, might lose house, anxious. Pain from previous assault. Denied suicide/self-harming thoughts*’.

(After-hours clinic presentation, entered by Clinic Manager)

**File excerpt-female patient aged 18–24:**

‘*Interaction prior to coded interaction (total 5 interactions):* (1) *intention to self-harm* [not coded], *police involved*; (2) *DV* [domestic violence] *assault*; (3) *DV assault*; (4) *disclosure of sexual assault*; (5) *DV assault, assistance to leave community*’.

(After-hours and clinic-hours presentations, entered by various clinic staff, including AHW, RAN, and GP)

Coded presentation (two weeks later):

‘*Separated from a violent partner, he has been at house threatening her, grandmother away; isolated; distressed, attempted to hang herself; found by community member standing on a crate with a noose around her neck*’.

(After-hours presentation, entered by GP)

#### 3.2.4. Services and Stakeholders

While there is documented engagement with specialist mental health services for the reviewed patients across all categories (Table 5), the features of the specialist engagement were markedly different across categories. Within Category A, specialist engagement was largely limited to an after-hours phone call from the Clinic GP or RAN to the KMHDS on-call psychiatrist. This appeared routine practice if a patient presented with suicidal ideation or after an attempted suicide. There were limited examples of follow-up of these patients, despite four patients being transferred from the community to the Broome Health Campus (hospital) due to their mental health presentation.

For Category C patients, all but one patient was having regular (2–4 weekly) face to face visits on the community with a KMHDS mental health support or social worker. For these patients, there is evidence of regular psychiatric review, ongoing medication review, and regular communication between KMHDS and the ACCHS.

Very few other stakeholders or services were identified through the file review as having a role in psychosocial or therapeutic care.

#### 3.2.5. Clinic-Based Response Summary

All reviewed files showed evidence of either a situational crisis response and/or an ongoing medication management plan. Most files (20/30) demonstrated ongoing clinic engagement with regards to a patient’s mental health/distress. Engagement was commonly documented as an assessment of the patient’s current home and family situation, mental health screening (generally not via a validated tool), brief intervention/psychosocial support, and/or medication. There was limited evidence of formal therapeutic interventions occurring within the clinic, and it is noted that no clinics included in the review had social and emotional wellbeing workers, counsellors, or psychologists on staff during 2020. For those files where follow-up was not recorded (10/30), there was evidence of patients not attending follow-up appointments with the clinic or attending but not wishing to discuss distress/mental health. There were also instances where follow-up only documented physical health matters.

For patients with a diagnosis of schizophrenia, the majority of interactions documented in MMEx were in relation to medication adherence. These patients were taking multiple medications, including an anti-psychotic injection either fortnightly or monthly, and the ACCHS had a primary role in locating and engaging with the patient to administer their medication. Often clinic staff needed to manage a patient’s medication hesitancy. Frequent home visits and engagement of family were noted as key methods to promote medication adherence. Two patients actively decreased their medication regime during the audit period. Two patients were admitted to a specialist mental health ward during the review period; the remainder were actively managed in the community despite challenges relating to hallucinations, auditory commands, and aggression. Notes demonstrate the role of carers and clinical relationship with carers/family in helping to monitor and manage patients. Carer fatigue and need for carer respite were also documented.

## 4. Discussion

This research has demonstrated that the remote ACCHS included in this study are responding to patient presentations of mental ill-health and psychological distress as an ongoing feature of clinical care. Yet there are significant barriers to diagnosis and effective treatment. The results of this study indicate that interaction between the ACCHS and a patient increases when the patient is experiencing significant risks or stressors, and/or if chronicity is a factor. These findings are congruent with other Aboriginal and Torres Strait Islander mental health research that articulate the role of psychosocial risks, many related to the social determinants of health [22], in the presentations of Aboriginal distress [12,23,24,25,26]. Violence within families, interpersonal conflict, and insecure or inappropriate housing were common antecedents, or attendants, to mental health presentations. These factors, along with the complex health profiles [19] and a preponderance of drug and alcohol use, provide a context to the challenges faced by patients. This context illustrates both the importance and limitations of clinical care in optimizing patient wellbeing.

At a population level, the most common mental health conditions for First Nations Australians are depression and anxiety [27]. This study found a limited diagnosis of these disorders. Diagnosis of a mental health condition was more frequently associated with the low prevalence disorder of schizophrenia. Workforce hesitancy in engaging and screening patients about their experiences of distress is a likely contributor to this result [28,29]. Improving workforce education and ensuring the availability of appropriate screening tools would likely see an increase in the numbers of patients diagnosed with common mental health disorders, such as depression and stress [30,31,32].

Screening for and diagnosis of mental health problems is only useful to the patient if it helps inform a pathway to support and recovery. The Australian health policy standard is for the GP to work with a patient to create a mental health care plan [27]. Typically, the GP and specialist services work together to support patient wellness. The file review exposed an overwhelming absence of therapeutic or social support services involved in patient care. Given the nature of the study, we are unable to conclude if this represents barriers with healthcare professionals referring patients for further support [29], the limited availability of services in remote communities [29,33], or challenges with clinical record keeping. Importantly, we are unable to discern if the lack of referrals is commensurate with patient desire, perhaps due to the pervasive stigma associated with mental health or the presence of cultural supports that are more responsive to a patient’s needs. Cultural supports could include family members, access to Elders, or other traditional lore or cultural practices [34,35,36,37]. It is hoped that subsequent qualitative work will help animate a more complete understanding of the interaction between remote ACCHS and the provision of mental health care [38].

Our findings highlight the importance of adopting a standardized approach to the clinical coding of presentations related to mental health. This approach is currently not implemented in Kimberley health services [5] and represents an important gap in service delivery. Moving towards standardized clinical coding will help the clinics to identify the extent and particulars of mental health presentations. It would allow for monitoring of trends or changes in mental health-related presentations at the clinic level [39]. This could help identify what resources may be required within the clinics to help respond to these presentations and act as a baseline to measure the benefits of any future mental health interventions that take place within the clinic or broader community [39].

The burden of chronic psychiatric disorders evident in the file reviews is consistent with other First Nations research [40]. This study contributes to the literature [41] by showing that the reviewed patients are largely managed in their very remote communities, with care shared between the ACCHS and KMHDS. Only two of the ten patients with chronic psychiatric disorders reviewed had one or more episodes of hospitalization during the study period, which is viewed as a proxy for the success of the community-based management.

The audit and related file review occurred during the first wave of COVID-19. The Western Australian Government enacted legislation to restrict movement in and out of remote communities for several months in an attempt to keep Aboriginal residents safe from the spread of COVID-19 [42]. It is unknown what impacts this had on people’s mental health [43]. While clinics remained operational during this time, it is noted that COVID-19 may have impacted the number of community members presenting to the clinic. Another limitation of this study is the use of clinical coding to define the number of patients who had mental health-related interactions with the clinics [44]. Clinical coding makes auditing efficient, but it is noted that if a clinician did not code using the terms included in our search parameters, then this information was excluded from the audit. Given the diversity of coded terms that the file review exposed, it is likely that this audit significantly underrepresents the number of Aboriginal patients who are interacting with ACCHS in response to their mental health. Despite these limitations, this study is the first of its kind for the Kimberley region. The study was completed with Aboriginal and non-Aboriginal researchers and helps quantify the essential role that ACCHS have in responding to psychological distress and mental ill-health in a remote context. To achieve healthcare equity for remote Aboriginal patients, there is a need to understand the complexities of real-world mental health service delivery; the findings from this research allow services to identify barriers to effective and culturally secure mental health care. The findings from this research can now be used to help inform real-world next steps for the remote Kimberley ACCHS in moving towards a model of care that is safe and responsive to patients’ presentations of mental ill-health and psychological distress. Our results show that a standardized approach to clinical coding for mental health presentations and the upskilling of health professionals to ensure they are confident and consistent in asking patients about their mood, psychosocial context, and current stressors are key elements in enhanced mental health model of care. Given the limited access and availability of external support services across the Kimberley, the model of care would need to determine ways to ensure the provision of brief intervention and ongoing psychosocial care of patients is embedded within existing clinic resources and structures [45,46,47].

## 5. Conclusions

Improving the mental health and wellbeing of First Nation Australians is a national priority. This data helps to quantify the preponderance and nature of distress and mental ill-health in remote primary health care settings. This, in turn, helps contextualize pathways for internal service improvements and broader advocacy to ensure culturally safe mental health care. Embedding mental health models of care in the provision of overall primary health care for First Nations people is an achievable and necessary component in improving mental health and wellbeing.

## Figures and Tables

**Table 1:** Patient and mental health interaction characteristics.

Patient and Interaction Characteristics		n	(%) ^a^
**Patients**		**92**	
Sex			
	Male	47	(51)
	Female	45	(49)
Age (years) ^b^			
	18–24	27	(29)
	25–34	30	(33)
	35–44	19	(21)
	45–54	12	(13)
	55 and over	4	(4)
Clinic ^c^			
	Clinic 1	27	(29)
	Clinic 2	12	(13)
	Clinic 3	53	(58)
Number of interactions			
	1	41	(45)
	2–9	37	(40)
	≥10	14	(15)
**Clinically coded interactions ^d^**		**403**	
Clinic			
	Clinic 1	192	(48)
	Clinic 2	28	(7)
	Clinic 3	183	(45)
Category of interaction/presentation			
	Did not attend appointment- mental health	11	(3)
	Mental health review	19	(4)
	Mental health medication	8	(2)
	Mental health assessment	3	(1)
	Mental health problem	110	(27)
	Mental health disorder	153	(38)
	Mental disability	8	(2)
	Mental disease	4	(1)
	Mental illness	30	(7)
	Mental distress	12	(3)
	Mental health crisis	1	(0)
	Self-harm	5	(1)
	Suicide attempt	15	(4)
	Suicidal ideation	13	(3)
	Suicide threat/fear	8	(2)
	Suicide plan	3	(1)

^a^ Percentages may not add up to 100 due to rounding. ^b^ Age at 3 February 2020. ^c^ Clinic 3 refers to a cluster of three very remote clinics. Seven patients attended two different clinics within this cluster. ^d^ Interactions relating to mental health indicated by progress notes that included the terms “mental *”, “self-harm”, or “suicid *”. Similar terms have been combined into categories. All instances of “mental disability” were confirmed to relate to mental health.

**Table 2:** Demographics for 30 file review patients.

File Review Patient Characteristic	Patient Category Based on Number of Coded Mental Health-Related Interactions ^a^			
	A (1 Coded) *n* = 10	B (2–9) *n* = 10	C (≥10) *n* = 10	Total *n* = 30
Sex				
Male	5	5	8	18
Female	5	5	2	12
Age (years)				
18–24	3	5	2	10
25–34	2	2	5	9
35–44	3	3	1	7
45–54	2	0	2	4
Clinic				
Clinic 1	3	4	5	12
Clinic 2	3	2	0	5
Clinic 3	4	4	5	13

^a^ Interactions relating to mental health indicated by progress notes in 2020 that included the terms “mental *”, “self-harm”, or “suicid *”.

**Table 3:** Median number of mental health-related interactions in 2020 based on file reviews, with comparison to audit, for 30 patients.

Mental Health-Related Interactions in 2020 Using Different Criteria	Patient Category Based on Number of Coded Mental Health-Related Interactions ^a^			
	A (1 Coded) *n* = 10	B (2–9) *n* = 10	C (≥10) *n* = 10	Total *n* = 30
Median interactions using audit coding criteria (IQR) ^a^	1 (1–1)	2.5 (2–4)	17.5 (15–18)	2.5 (1–15)
Median interactions in file reviews (IQR) ^b^	5 (2–6)	8.5 (3–19)	69 (62–96)	10.5 (5–62)

^a^ Interactions relating to mental health indicated by progress notes that included the terms “mental *”, “self-harm”, or “suicid *”. ^b^ Interactions indicated by progress notes in which the content related to mental health condition, mental health medication, suicide, self-harm.

**Table 4:** Characteristics of first mental-health coded presentation for 30 file review patients.

Characteristics of First Coded Presentation 2020	Patient Category Based on Number of Coded Mental Health-Related Interactions ^a^			
	A (1 Coded) *n* = 10	B (2–9) *n* = 10	C (≥10) *n* = 10	Total *n* = 30
Timing				
Clinic hours	4	4	10	18
After hours	6	6	0	12
Coding				
Suicide (intentions, threatening, ideation)	3	1	1	5
Suicide attempt	1	3	0	4
Self-harm	3	1	0	4
Mental health medication administered	0	1	0	1
Mental health (problem, disorder, impairment)	2	4	7	13
Mental distress	1	0	1	2
Major mental health episode	0	0	1	1

^a^ Interactions relating to mental health indicated by progress notes in 2020 that included the terms “mental *”, “self-harm”, or “suicid *”.

## Data Availability

Data cannot be shared publicly because of the ethical restrictions and data sovereignty considerations involved in working with a small population of Aboriginal people in Western Australia. Indigenous data sovereignty is defined as the right of Indigenous peoples to determine the means of collection, access, analysis, dissemination, and reuse of data pertaining to the Indigenous peoples from whom it relates or was derived. As such, the minimal dataset will be made available upon request for researchers who meet the criteria for access to confidential data. Interested researchers should contact Emma Carlin at Emma.Carlin@rcswa.edu.au for data requests.
